# Iron Homeostasis and Hepcidin Concentration in Patients With Acromegaly

**DOI:** 10.3389/fendo.2021.788247

**Published:** 2022-02-08

**Authors:** Aleksandra Krygier, Ewelina Szczepanek-Parulska, Maja Cieślewicz, Elżbieta Wrotkowska, Justyna Chanaj-Kaczmarek, Marek Ruchała

**Affiliations:** ^1^Department of Endocrinology, Metabolism and Internal Medicine, Poznan University of Medical Sciences, Poznan, Poland; ^2^Department of Pharmacognosy, Poznan University of Medical Sciences, Poznan, Poland

**Keywords:** hepcidin, iron, erythropoiesis, acromegaly, complete blood count

## Abstract

Hepcidin is a protein responsible for maintaining iron (Fe) homeostasis. Data regarding the role of hepcidin in the pathomechanism of Fe balance disturbances associated with acromegaly (AG) are scarce. The aim of the study was to assess the impact of alterations in complete blood count parameters, Fe homeostasis, gonadal status and GH/IGF-1 on the level of hepcidin in AG patients. The study evaluated the differences in hepcidin concentration and iron homeostasis between patients newly diagnosed with AG in comparison to healthy control subjects (CS). We prospectively enrolled 25 adult patients newly diagnosed with AG and 25 healthy volunteers who served as CS. The level of hepcidin was measured using the Hepcidin 25 (bioactive) hs ELISA, which is a highly sensitive enzyme immunoassay for the quantitative *in vitro* diagnostic measurement (DRG Instruments GmbH, Germany). The median of hepcidin concentration in the serum of patients with AG was significantly lower 9.8 (6.2–18.2) ng/ml as compared to CS 21.3 (14.3–34.0) ng/ml (p = 0.003). In the AG group, a statistically significant negative correlation between hepcidin and IGF-1 (rho = −0.441) was observed. Our study demonstrated a decreased hepcidin level in AG patients in comparison to CS what may have a potentially protective effect against anemia through an increased bioavailability of Fe. Additionally, GH may have a positive direct or indirect effect on erythropoiesis. Further studies on larger patient groups are necessary in order to clarify the exact role of hepcidin in the regulation of erythropoiesis in the excess of GH/IGF-1.

## Introduction

Hepcidin constitutes an acute phase liver-derived protein, responsible for maintaining iron (Fe) homeostasis through both local and systemic impact (1, 2). Hepcidin triggers the internalization and degradation of ferroportin (the major Fe transporter) which results in a decrease in serum Fe level (3). Therefore, hepcidin level reflects the erythropoietic status of the organism, as erythrocyte production is dependent on the bioavailability of Fe. Iron, in turn, is a crucial ion for heme synthesis, which is a key hemoglobin (HB) component, essential for red blood cells (RBC) production. Iron metabolism is controlled by hepcidin on three levels: absorption in the duodenum, release from macrophages, and from hepatocytes (2). Furthermore, hepcidin is regulated by serum Fe level and hepatic Fe stores, representing a homeostatic feedback loop (4–6). There are also other stimulating factors, such as inflammation, autoimmune diseases, chronic kidney diseases (4, 7). Conversely, hemolysis, hemorrhage, anemia, and erythropoietin inhibit hepcidin, which enables the restoration of Fe resources and maintains effective erythropoiesis. Alterations in hepcidin level results in severe disturbances—excessive hepcidin causes Fe deficiency anemia, whereas its deficit leads to Fe overload, or hemochromatosis. Although hepcidin was first identified in 2000 (8), it has recently been evaluated in view of the various endocrine disorders associated with an increase in acute-phase proteins or with concomitant disturbances in Fe homeostasis (9–11). As a result of such reports, a growing awareness of the hormonal influence on Fe metabolism and hepcidin levels has emerged.

Acromegaly (AG) is a rare disease caused by excessive production of the growth hormone (GH) and insulin-like growth factor 1 (IGF1) (12). In more than 90% of cases, the disorder stems from a GH-secreting pituitary adenoma (13, 14), and as a consequence, patients present with somatic growth, metabolic dysfunctions (15, 16), and a decreased quality of life (17). The stimulatory effect of GH on the mammalian erythropoiesis *in vitro* has been widely recognized (18, 19). In fact, Hanley et al. confirmed the promoting effect of both GH and IGF-1 on the hematopoietic colony forming cells in human fetal bone marrow (20). In another study, a decrease in the amount of the hematopoietic bone marrow cells caused by aging observed in rodents was found to be reversed by GH administration, through an increase in the myeloid colony forming units (21). Moreover, Tsarfaty et al. demonstrated *in vivo* the stimulation of the hematopoietic progenitor cells in bone marrow following the administration of human recombinant IGF-1, and also its promoting impact on erythroid precursor cells (22). Moreover, the stimulation of erythroid cells by GH and IGF-1 has been demonstrated not only in the bone marrow but also in the peripheral blood (19). Two other previous studies revealed GH involvement in erythropoiesis, showing a significant improvement in erythropoiesis indices which occurred in the course of GH therapy in children suffering from GH deficiency (23, 24), although the abovementioned tendency was also observed in adults (25). In fact, insulin-like growth factor 1 is believed to be a key growth-promoting mediator of GH on human erythroid precursors (26, 27). Despite the recognized involvement of GH in the stimulation of erythropoiesis and altered blood count parameters in AG patients, data regarding the role of hepcidin in the pathomechanism of Fe homeostasis disturbances associated with AG are still scarce.

The objective of the study was to assess the impact of alterations in complete blood count parameters, Fe homeostasis, gonadal status and GH/IGF-1 on the level of hepcidin in AG patients. To the best of our knowledge, this has been the first study evaluating the difference in hepcidin concentration between the newly diagnosed AG patients and the healthy control subjects (CS).

## Materials and Methods

The study was prospective and observational. We enrolled 25 adult patients with newly diagnosed AG. The CS comprised 25 healthy volunteers, matched both for age and gender. The diagnosis of AG was based on the following factors: characteristic clinical signs and symptoms, laboratory tests (IGF-1 level above the reference range for age and gender, and GH level >0.4 ng/ml measured by ultrasensitive assay in 75 g oral glucose tolerance test) and a confirmed pituitary adenoma in magnetic resonance imaging—MRI (12). The strict exclusion criteria for both groups were as follows: anemia, hemolysis, hemorrhage, neoplastic process, autoimmune or inflammatory diseases, chronic kidney or liver diseases, pregnancy or breast-feeding, hemochromatosis, the use of erythropoietin, exogenous supplementation (iron, vitamin B12, folic acid), or surgical therapy (within the previous 6 months).

All patients agreed to participate in the study and signed a written informed consent. The research was conducted in accordance with the Declaration of Helsinki, and was approved by the Poznan University of Medical Sciences Bioethics Committee (approval number: 176/17).

Extensive laboratory assessment was performed in both groups. The level of hepcidin was measured using the Hepcidin 25 (bioactive) hs ELISA, a highly sensitive enzyme immunoassay for the quantitative *in vitro* diagnostic measurement (DRG Instruments GmbH, Germany). Complete blood count parameters were evaluated by an automated flow cytometer Sysmex-XN 1000 (Sysmex Europe GmbH, Born-barch, Germany). In order to fully investigate Fe homeostasis, ferritin level was evaluated in Hitachi Cobas e601 chemiluminescent analyzer (Roche Diagnostics), whereas Hitachi Cobas e501 analyzer (Roche Diagnostics) was used to analyze Fe levels. Thyroid gland status was assessed by measuring thyroid-stimulating hormone (TSH), free triiodothyronine and thyroxine (fT3, fT4), anti-thyroid peroxidase antibodies (aTPO), and anti-thyroglobulin antibodies (aTG) using Hitachi Cobas e601 chemiluminescent analyzer (Roche Diagnostics).

Other potential factors, and also chronic kidney and liver diseases, were excluded on the basis of alanine aminotransferase (ALT), aspartate aminotransferase (AST), and C-reactive protein (CRP) measurements using Hitachi Cobas e501 analyzer (Roche Diagnostics). The estimated glomerular filtration rate (eGFR) was assessed by an online medical calculator (https://www.mdcalc.com/mdrd-gfr-equation) based on the MDRD (Modification of Diet in Renal Disease Study) equation.

In terms of the AG patients, GH, IGF-1, prolactin, testosterone and estradiol, and also thyroid (TSH, fT4, fT3) and adrenal function (ACTH, cortisol) were analyzed by Hitachi Cobas e601 chemiluminescent analyzer (Roche Diagnostics). The IGF-1 norm was applied with respect to age and gender, and was presented as a percentage above of the upper normal range for an individual (%IGF-1). In turn, GH was assessed in oral glucose tolerance test after the administration of 75 g of glucose, whereas GH concentration for patients with diabetes was determined as a mean value from 5 separate measurements in 30-minute intervals (GH five-points profile). Moreover, the volume of pituitary adenoma was evaluated in MRI. A standardized SAGIT questionnaire was completed in all AG patients prior to treatment (where the SAGIT acronym denotes: Signs and symptoms (0–4 points), Associated co-morbidities (0–6 points), GH level (0–4 points), IGF-1 level (0–3 points) and Tumor profile (0–5 points) (28). Additionally, the concomitant secondary thyroid and adrenal insufficiency constituted the exclusion criteria.

Statistical analysis was performed using STATISTICA software (StatSoft, Tulsa, Oklahoma, USA) and MedCalc Software (MedCalc Software Ltd, Ostend, Belgium). In most cases, a Shapiro–Wilk test did not reveal the normal distribution for the analysed data, thus, only non-parametric tests were applied, and therefore all values are expressed as median and 25–75% interquartile range (IQR). Furthermore, we performed Mann–Whitney U-test in order to compare the AG and CS groups. In terms of hepcidin and the parameters related to erythropoiesis, the effect size (η2) was evaluated. Spearman’s rank correlation coefficient was applied to evaluate hepcidin concentration and all the investigated laboratory parameters. Parameters with different gender-dependent reference values were calculated separately (RBCs, HCT, HB, Fe, and ferritin). The level of statistical significance was established at p <0.05.

## Results

The study comprised 25 adult patients with newly diagnosed AG. The CS included 25 healthy volunteers matched for age and gender. Gender distribution in both groups was as follows: men (n = 16) and women (n = 9). The AG patients were aged between 22 and 80 years (49 ± 17 years), whereas the CS subjects were aged between 28 and 76 years (58 ± 12 years). We found a slight difference in age between the two groups (p = 0.04), although a correlation between hepcidin and age was not observed.

### Hepcidin

**Figure 1:**
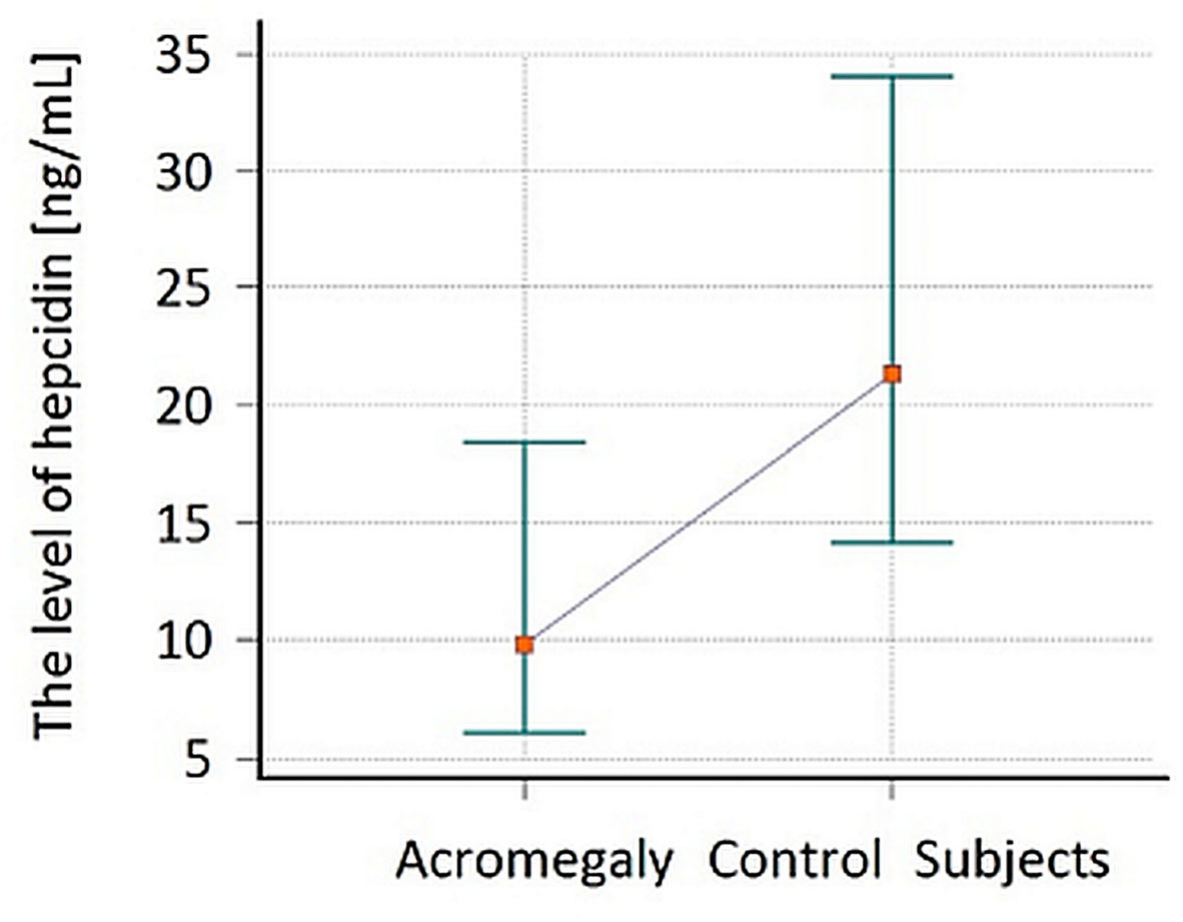
The comparison of hepcidin level in patients with acromegaly (AG) at the time of the diagnosis and in the control subjects (CS). Values are expressed as median and interquartile range (IQR).

**Figure 2:**
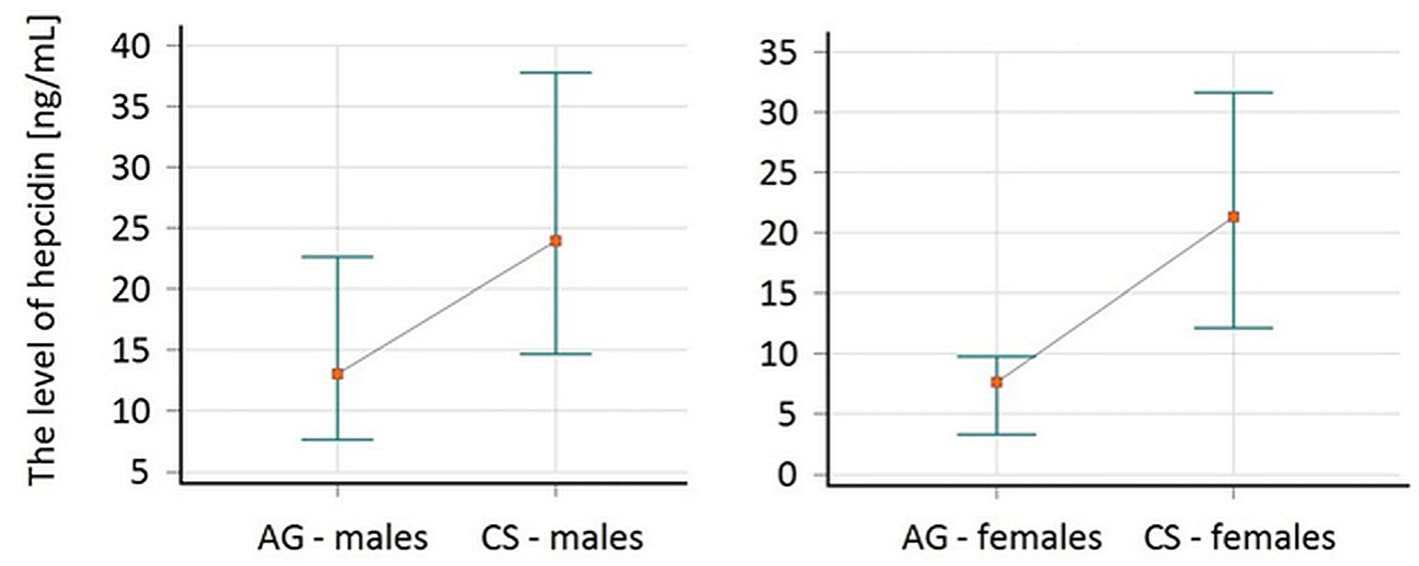
The comparison of hepcidin level in patients with acromegaly (AG) at the time of the diagnosis and in the control subjects (CS) divided by gender. Values are expressed as median and interquartile range (IQR).

The median (IQR) of hepcidin concentration in the serum of patients with AG amounted to 9.8 (6.2–18.2) ng/ml, and was statistically significantly lower (p = 0.003) in comparison to CS 21.3 (14.3–34.0) ng/ml **(****Figure 1****)**. The effect size (η2) for this group was equal to 0.180 which in interpretation according to Cohen (1988) indicated a large effect, whereas in the interpretation according to Hattie (2009) represented the zone of desired effects. The median of hepcidin in AG women was 7.6 (3.5–9.7) ng/ml in comparison to CS women 21.3 (14.3–30.9) ng/ml, which was at the limit of statistical significance (p = 0.09), and η2 for this group was equal to 0.165. In the group of men, the difference in median of hepcidin reached statistical significance [p = 0.03, 13.0 (7.6–22.6)_AG_ vs. 23.9 (14.6–37.8)_CS_ ng/ml, η2 = 0.155]. The comparison of hepcidin level in the subgroups divided according to gender was presented in **Figure 2**.

Spearman’s rank correlation was used to evaluate hepcidin and all blood parameters analysed in this study, taking into consideration the division according to gender. In the AG group, the calculated coefficient indicated statistically significant (p <0.05) negative correlations between hepcidin and IGF-1 (rho = −0.441), as well as RBC in men (rho = −0.597). Furthermore, a positive correlation (p <0.05) was found between hepcidin and ferritin in men (rho = 0.700). An equivalent analysis was performed in terms of CS, and a statistically significant correlation between hepcidin and ferritin in both genders [male (rho = 0.682); female (rho = 0.867)] was observed.

### Other Parameters

The pituitary function was assessed only in the group of patients with AG. Since the level of IGF-1 depends on age and gender, the percentage over the upper limit of the normal range was calculated. In the group of 16 men, the median of testosterone was 7.1 (4.5–9.9) nmol/L, where the normal range with regard to the total testosterone is 9.9–27.8 nmol/L. Twelve of the patients presented with hypogonadism due to pituitary gland adenoma and in the rest (4 of 16 men) we observed a correct activity of the pituitary–gonadal axis. Despite normal gonadotropin secretion, the median levels of testosterone were in the lower reference range. Moreover, 7 out of 9 women were in postmenopausal period and the level of estradiol was below 5 pg/ml. A non-significant correlation between the level of hepcidin and testosterone or estradiol was noted [Spearman’s rank correlation between hepcidin and testosterone was rho = −0.594 (p >0.05), and for estradiol rho = −0.365 (p >0.05)]. Additionally, the median level of prolactin was 278 (IQR 219–393) µIU/ml in women and 379 (240–648) µIU/ml in men. One female patient presented a higher value of prolactin; nevertheless, she was in the postmenopausal period, whereas a mild hyperprolactinemia was observed in 7 out of 16 male subjects **(****Table 1****)**.

**Table 1:** The parameters evaluated in acromegaly group.

Parameter	Reference range	Acromegaly group AG	Control subjects CS
IGF-1 (ng/ml)	norm for age and gender	775 (675–959)	–
%IGF-1	% of normal range for age and gender	304 (220–345)	–
GH/test^a^ (ng/ml)	<0.4	7.4 (5.1–21.5)	–
GH/profile^b^ (ng/ml)	<2.5	14.6 (4.8–17.2)	–
V (mm^3^)	–	2,040 (572–4,725)	–
SAGIT	0–26	13 (11–15)	–
Testosterone in men (nmol/L)	9.9–27.8	7.1 (4.5–9.9)	–
Prolactin (µIU/ml)	70–510^c^	278 (219–393)	–
	85–390^d^	379 (240–648)	–
Hepcidin (ng/ml)	0.2–47.7	9.8 (6.2–18.2)	21.3 (14.3–34.0)

Values are expressed as median (IQR).

IGF-1, insulin-like growth factor 1; GH, Growth Hormone; V, volume of pituitary adenoma; SAGIT, instrument is multidimensional, comprising five sections that assess key features of acromegaly: signs and symptoms (S), associated comorbidities (A), GH levels (G), IGF-1 levels (I), and the Tumor profile (T).

a In oral glucose tolerance test.

b Five-point profile of growth hormone.

c Female.

d Male.

### Comparisons

A comparison of both AG and CS groups revealed significant differences (provided in **Table 2**). The median ferritin in men with AG 94.0 (59.0–142.0) ng/ml was statistically significantly decreased (p = 0.02) as compared to CS 184.0 (112.5–245.0) ng/ml. The parameters of thyroid, liver, kidney or inflammation potentially influencing hepcidin level did not present significant differences.

**Table 2:** Biochemical parameters in patients with AG at the moment of diagnosis compared to CS.

Parameter	Reference range	Acromegaly (AG)	Control subjects (CS)	p-value	Eta squared (η2)
WBC (×10^3/^μl)	3.9–11.0	6.4 (5.5–7.6)	6.7 (6.3–8.0)	NS	
*RBC—m (×10^6/^μl)	3.2–5.4	5.0 (6.7–5.1)	4.9 (4.8–5.0)	NS	0.008
*RBC—f (×10^6/^μl)	3.5–5.2	4.3 (4.3–4.8)	4.7 (4.6–4.8)	NS	0.085
*Hb—m (g/dl)	14.0–18.0	14.9 (14.4–15.4)	15.0 (14.4–15.5)	NS	0.004
*Hb—f (g/dl)	**12.0–15.6**	**13.4 (12.8–13.8)**	**14.7 (14.0–15.0)**	**p = 0.02**	**0.328**
*HTC—m (%)	40.7–50.3	43.2 (41.4–49.9)	43.7 (42.4–45.3)	NS	0.022
*HTC—f (%)	**36.1–44.3**	**39.4 (39.2–39.4)**	**43.0 (42.7–43.5)**	**p = 0.02**	**0.293**
MCV (fl)	80.0–99.0	89.3 (85.6–91.8)	90.9 (87.5–93.4)	NS	
MCH (pg)	27.0–33.5	30.2 (29.5–31.9)	30.9 (27.7–31.6)	NS	
MCHC (g/dl)	31.0–38.0	34.3 (33.8–34.8)	34.1 (33.6–34.7)	NS	
RDW-CV (%)	11.0–16.0	13.0 (12.4–13.5)	13.0 (12.5–13.4)	NS	
PLT (fl)	130–400	246 (214–265)	242 (219–289)	NS	
PDW (fl)	9.0–17.0	11.9 (10.8–13.4)	12.0 (11.3–13.8)	NS	
MPV (fl)	9.0–13.0	10.5 (9.7–11.1)	10.4 (10.0–11.2)	NS	
P-LCR (%)	13.0–43.0	28.3 (23.2–33.9)	28.6 (24.8–35.2)	NS	
*Ferritin—m (ng/ml)	**30–400**	**94 (59–142)**	**184 (112–245)**	**p = 0.02**	**0.190**
*Ferritin—f (ng/ml)	20–200	36 (22–99)	109 (44–188)	NS	0.219
*Fe—m (μg/dl)	50–158	124 (98–190)	137 (99–160)	NS	0.001
*Fe—f (μg/dl)	37–145	147 (98–152)	100 (89–145)	NS	0.085
CRP (mg/L)	<5.0	0.3 (0.3–0.6)	1.1 (0.6–2.4)	NS	
ALT (U/L)	10–41	17 (14–22)	22 (15–29)	NS	
AST (U/L)	10–37	20 (15–23)	18 (14–24)	NS	
TSH (μIU/ml)	0.2–4.2	1.3 (0.9–1.6)	1.4 (0.95–1.7)	NS	
fT3 (pmol/L)	3.9–6.7	5.2 (5.0–5.7)	5.1 (4.7–5.5)	NS	
fT4 (pmol/L)	11.5–21.0	16.4 (14.4–19.4)	17.7 (16.2–18.1)	NS	
aTPO (IU/ml)	<34	11 (9–22)	12 (9–15)	NS	
aTG (IU/ml)	10–115	11 (10–18)	10 (10–11)	NS	
eGFR		113 (94–128)	96 (83–118)	NS	

Values are expressed as median (IQR) for non-parametric tests (Mann–Whitney U-test).

NS, non-significant.

*Parameters with different reference range in men and women; m, male; f, female.

WBC, white blood cells, RBC, red blood cells; HGB, hemoglobin; HCT, hematocrit; MCV, mean corpuscular volume; MCH, mean corpuscular hemoglobin; MCHC, mean corpuscular hemoglobin concentration; RDW-CV, red blood cell distribution width-coefficient of variation; PLT, platelets; PDW, platelet distribution width; MPV, mean platelet volume; P-LCR, platelet larger cell ratio; CRP, C-reactive protein; ALT, alanine aminotransferase; AST, aspartate aminotransferase; TSH, thyroid-stimulating hormone; fT3, free triiodothyronine; fT4, free thyroxine; aTPO, anti-thyroid peroxidase antibody; aTG, anti-thyroglobulin antibody; Fe, iron; e-GFR, estimated glomerular filtration rate. Bold values are statistically significant (p value < 0.05).

### Correlations

All parameters in both groups involved in the study were correlated; therefore, we presented the most significant correlations. In both study groups (AG and CS) we observed a positive, statistically significant correlation between the mean corpuscular volume (MCV) and mean corpuscular hemoglobin (MCH) (rho = 0.912_AG_ vs. 0.713_CS_), and also RBC and hematocrit (HTC) (rho in men 0.685_AG_ vs. 0.705_CS_, rho in women 0.748_AG_ vs. 0.667_CS_). In the male group, we demonstrated also a correlation between RBC and HB (p <0.05, rho = 0.686_AG_ vs. 0.580_CS_), whereas in the female group, a correlation with HB and HTC (p <0.05, rho = 0.752_AG_ vs. 0.850_CS_) was found.

In the male AG group of patients we demonstrated a statistically significant correlation between RBC and MCV (rho = −0.630), RBC and MCH (rho = −0.662), RBC and ferritin (rho = −0.668), MCV and ferritin (rho = 0.702), MCH and ferritin (rho = 0.750), and also HB and HTC (rho = 0.885). In terms of the female subgroup correlations (p <0.05) between RBC and MCV (rho = −0.900) as well as RBC and MCH (rho = −0.850) were observed. Furthermore, in the AG group, a statistically significant correlation was noted between IGF-1 and SAGIT (rho = 0.426), %IGF-1 and Fe in men (rho = 0.532), as well as %IGF-1 and TSH (rho = 0.428).

## Discussion

To the best of our knowledge, this has been the first study which prospectively evaluated differences in hepcidin concentration between the newly diagnosed AG patients and healthy CS. Moreover, we profoundly assessed the impact of alterations in complete blood count parameters, Fe homeostasis, gonadal status and GH/IGF-1 on the level of hepcidin in AG patients.

In all patients with newly diagnosed AG, and following the gender subdivision, we observed a decreased level of hepcidin in comparison to the CS. In fact, a median serum hepcidin concentration was decreased more than two times in the AG group than in the CS. A potential explanation of the observed change includes an impact of elevated GH, and consequently IGF-1 (29), which constitute crucial pathogenetic factors in the development of AG, and significantly affect hepcidin concentration levels and Fe homeostasis. According to the study by Troutt et al. in 2012, a reduction of hepcidin level following GH administration in the healthy volunteers was found, which was presumably due to the stimulating effect on erythropoiesis (30) reported in the previous studies (18, 19, 27).

Considering the role of hepcidin, a decrease in this protein in the AG group should potentially cause a greater bioavailability of iron and ferroportin, resulting in an increase in erythropoiesis. However, it was not observed; in fact, the pro-erythropoiesis parameters (ferritin, iron) were either the same or lower in the AG group compared to the CS group. Moreover, we did not observe statistically significant differences in the complete blood count between AG and CS, except HB and HTC in female patients. Both aforementioned parameters were lower in the AG group than in the CS group, nevertheless, they were still within the normal range with the simultaneous normal level of Fe and ferritin.

An example of a special situation, where hepcidin is inhibited or low, while there is no iron overload in the blood, is pregnancy. Van Santen et al. have noted a significant decrease of hepcidin since the second trimester of gestation that persists through the rest of the pregnancy (31). This trend was likely related to the increase of iron consumption by fetus growth, which could be recognized in the corresponding lowering in serum iron and erythropoietic parameters. We hypothesize that in acromegaly there might be a similar situation, and iron is intensively used for tissues and organs overgrowth. According to the current state of knowledge on iron homeostasis regulation, when tissue iron demands are high, hepcidin concentrations are low and vice versa (32–34). Such a situation takes place in patients with acromegaly. If the iron is required, it can be exported rapidly across the enterocyte basolateral membrane *via* ferroportin 1. It is then bounded to transferrin which distributes it around the body to the sites of utilization. Tissues with high proliferative capacity require large amounts of iron and express TfR1 on their plasma membrane. Thus, when iron supply becomes limited, its distribution to tissues becomes prioritized, possibly even at the expense of erythropoiesis. This may at least partially explain why we do not observed high Fe and erythrocyte indices in the blood of patients with AG.

The other possible explanation might be the direct influence of GH/IGF-1 on other regulators of iron homeostasis, i.e., suppression of divalent metal-iron transporter 1 (DMT1), responsible for nonheme iron import across the apical membrane of the enterocyte. The exact mechanisms of regulation of DMT1 are still not fully understood, however at least three possible mechanisms may be involved (translational regulation through IRE–IRP binding, interaction of PCBP2, degradation by ubiquitination depending on iron level) (35).

All patients participating in the study underwent both gastro- and colonoscopy at the time of the AG diagnosis to exclude active neoplasms and their influence on iron homeostasis parameters. According to Ward et al. and Xiang-Tao et al., the expression of hepcidin gene is enhanced in colorectal carcinogenesis, although it does not trigger systemic anemia. Low complete blood count parameters were associated with the increased colon cancer staging (36). Moreover, Xiang-Tao indicated that the expression of hepcidin depended on tumor stage (37). Therefore, it is possible to conclude that early stage lesions, such as polyps, are rather unlikely to interfere with our results.

The influence of GH and IGF-1 on erythropoiesis has already been demonstrated on multiple levels. The process of erythropoiesis depends on the production of RBCs which relies on the bioavailability of Fe—an important component of heme in HB, and also on the erythropoietin stimulating proliferation and differentiation of erythroid progenitor cells (29). GH and IGF-1 stimulate growth of erythroid precursor cells in the presence of erythropoietin. Additionally, GH receptors are present in the bone marrow (18), and IGF-1 receptors are located in erythrocytes. It has been hypothesized that the activity of GH is mainly mediated by IGF-1, which has a similar activity to erythropoietin and can act both directly, and also through the enhanced production of erythropoietin (23, 38, 39). In fact, Anttila et al. reported a positive correlation between serum IGF-1 and HB in healthy children (40). Furthermore, GH insufficiency patients develop anemia with low erythropoietin levels (20, 25). In contrast, Petrossians et al. did not observe any correlations between RBC (analysed separately for male and female patients) and GH in AG patients, although they noticed a limited statistical correlation between RBC count and IGF-1. Additionally, they also found no correlation of HB with GH, but observed a positive correlation with IGF-1 (41).

Fe concentration levels in both study groups in our research were similar. However, in the AG male group the level of ferritin (p <0.05) was twice as low as in the CS group, although still within the reference range. Ferritin, in turn, is a protein responsible for Fe storage and a marker of inflammation (42). According to the previous research, a decrease in ferritin levels has been linked to Fe deficiency (43). Decreased ferritin in AG, indicative of relative depletion in Fe stores, may potentially account for the decreased hepcidin observed in the AG group. In view of the pathophysiological mechanism of acromegaly, reduced blood count parameters (HB, HTC), together with Fe concentration within the normal limits, may suggest the utilization of Fe in other processes. This is presumably due to the previously mentioned increased Fe demand during organ overgrowth or the conversion of ribose nucleotides to deoxyribose nucleotides, which is an essential process for DNA replication and cell division (44), enhanced in AG (45).

The previously widely recognized positive correlation between hepcidin and ferritin (9–11), was repeatedly confirmed in our study, and was particularly evident in men. The recognized hepcidin suppressors include sex hormones (testosterone, 17β-estradiol, progesterone), and erythropoiesis [through ligands and/or modulators, such as erythroferrone (ERFE), growth differentiation factor 15 (GDF15), twisted-gastrulation 1 (TWSG1), and growth factors [hepatocyte growth factor (HGF), epidermal growth factor (EGF), platelet-derived growth factor BB (PDGF-BB)]. Additionally, Goodnough et al. noticed that IGF-1 and IGF-2 had no impact on hepcidin expression in the primary hepatocytes (46, 47). The well-defined regulatory pathways of hepcidin include the BMP-SMAD pathway (mediating the effect of Fe and inflammation on hepcidin), the IL-6 pathway (mediating the effect of inflammation), and the EPO–ERFE axis (mediating hepcidin suppression by erythropoietic activity) (46).

The AG patients and CS were euthyroid, with no autoimmune thyroid diseases and did not present with overt anemia, chronic liver or kidney disease and inflammation. The majority of male patients with acromegaly (75%) developed partial hypogonadotropic hypogonadism, whereas 78% women were in postmenopausal period, and/or suffered from the secondary gonadal failure. In the men from the CS group, the level of testosterone was not measured; however, it can be assumed that the studied groups were representative of each other, since the testosterone level in men tends to decrease with age, as confirmed by Feldman et al. (48). Most of the healthy women were in the postmenopausal age.

Previous clinical and experimental observations suggested that elevated levels of estrogen and testosterone may downregulate hepcidin synthesis (44). In 2012, Hou et al. investigated the biological effect of estrogen on Fe metabolism in the mouse model. Mice following ovariectomy presented a decreased HB and Fe serum concentrations, although tissue Fe level in the liver and spleen was increased. The transcription of hepatic hepcidin was elevated in the ovariectomized mice compared to the control mice, which confirmed that estrogen greatly contributes to Fe homeostasis by regulating hepatic hepcidin expression directly through a functional estrogen response element in the promoter region of hepcidin gene (49). Subsequently, Yang et al. observed an inhibition of hepcidin synthesis induced by 17β-estradiol, and an increased Fe uptake, a mechanism to compensate Fe loss during menstruation (50). Recent studies have confirmed that estrogen activity contributes to the changes in serum Fe status; a negative correlation between hepcidin and estrogen or Fe was demonstrated in menstruating women which is a mechanism increasing serum Fe content (51). The latest reports indicated that testosterone suppressed hepcidin (44), either through direct or indirect mechanisms, resulting in the increased Fe turnover and maintaining erythropoiesis during severe energy deficit (52). Furthermore, Guo et al. emphasized the negative impact of testosterone on hepcidin, which increases Fe availability and erythropoiesis. However, it was also demonstrated that hepcidin suppression was not essential for mediating effects of testosterone on erythropoiesis in healthy mice (53).

Therefore, in our study, we excluded the reducing effect of estrogen and testosterone on hepcidin levels in the AG group. As in the AG group, a decreased hepcidin level was noted, regardless of the gonadal function compared to the CS. We also observed a statistically significant negative correlation of hepcidin and IGF-1, as well as positive correlation of %IGF-1 and Fe in the male group. Thus, we may suggest that in patients with AG, the crucial influence on low hepcidin levels was the concentration of GH/IGF-1 and/or Fe homeostasis.

Another issue worth noting is the prolactin concentration in our group. In the literature the data with regard to the influence of prolactin levels on hepcidin concentration is scarce. We have found only one report on 40 serum samples from non-pregnant hyperprolactinemic women, where the authors noticed a decreased level of hepcidin in comparison to controls with normal prolactin concentration. This may constitute a limitation in our study, since we cannot exclude the effect of elevated prolactin levels on hepcidin (54). However, in our group only one woman and few men presented with only mild hyperprolactinemia. Patients with high prolactin levels (above 3 times the upper limit of the normal value) with a high probability of a mixed GH-prolactin secreting tumor, were not included in the analysis. Moreover, Wang et al. provided evidence that prolactin directly supresses hepcidin expression (54). Taking into account that prolactin and growth hormone are largely analogues in their chemical structure and active center (prolactin—peptide hormone with 199 amino acids, growth hormone—peptide hormone with 191 amino acids), function and origin from the anterior pituitary lobe (55, 56), we may assume that growth hormone may exert similar physiological effect on hepcidin.

In conclusion, our study demonstrates a decreased hepcidin level in AG patients in comparison to the CS group, which may have a potentially protective effect against anemia through an increase in the bioactivity of Fe. Additionally, GH may positively affect erythropoiesis, both directly and indirectly. Further studies on larger groups of patients are required to investigate the exact role of hepcidin in the regulation of erythropoiesis in the excess of GH/IGF-1.

## Data Availability Statement

The raw data supporting the conclusions of this article will be made available by the authors, without undue reservation.

## Ethics Statement

The studies involving human participants were reviewed and approved by the Bioethical Committee of Poznan University of Medical Sciences. The patients/participants provided their written informed consent to participate in this study.

## Author Contributions

AK, ES-P, and MR performed the study design. AK and ES-P performed data acquisition and interpretation. AK performed statistical analysis. EW and JC-K were involved in the laboratory measurements. AK, ES-P, and MC prepared the manuscript. ES-P and MR completed the final revision of the manuscript. All authors contributed to the article and approved the submitted version.

## Funding

The work was supported by the PRELUDIUM 12 grant from the Polish National Centre for Science (2016/23/N/NZ5/02573).

## Conflict of Interest

The authors declare that the research was conducted in the absence of any commercial or financial relationships that could be construed as a potential conflict of interest.

## Publisher’s Note

All claims expressed in this article are solely those of the authors and do not necessarily represent those of their affiliated organizations, or those of the publisher, the editors and the reviewers. Any product that may be evaluated in this article, or claim that may be made by its manufacturer, is not guaranteed or endorsed by the publisher.
